# Carbohydrate, but not fat, oxidation is reduced during moderate-intensity exercise performed in 33 vs. 18 °C at matched heart rates

**DOI:** 10.1007/s00421-023-05225-0

**Published:** 2023-05-18

**Authors:** Thanchanok Charoensap, Andrew E. Kilding, Ed Maunder

**Affiliations:** Sports Performance Research Institute New Zealand, Auckland University of Technology, Auckland, New Zealand

**Keywords:** Substrate metabolism, Cycling, Heat, Intensity

## Abstract

**Purpose:**

Exposure to environmental heat stress increases carbohydrate oxidation and extracellular heat shock protein 70 (HSP70) concentrations during endurance exercise at matched absolute, external work rates. However, a reduction in absolute work rate typically occurs when unacclimated endurance athletes train and/or compete in hot environments. We sought to determine the effect of environmental heat stress on carbohydrate oxidation rates and plasma HSP70 expression during exercise at matched heart rates (HR).

**Methods:**

Ten endurance-trained, male cyclists performed two experimental trials in an acute, randomised, counterbalanced cross-over design. Each trial involved a 90-min bout of cycling exercise at 95% of the HR associated with the first ventilatory threshold in either 18 (TEMP) or 33 °C (HEAT), with ~ 60% relative humidity.

**Results:**

Mean power output (17 ± 11%, *P* < 0.001) and whole-body energy expenditure (14 ± 8%, *P* < 0.001) were significantly lower in HEAT. Whole-body carbohydrate oxidation rates were significantly lower in HEAT (19 ± 11%, *P* = 0.002), while fat oxidation rates were not different between-trials. The heat stress-induced reduction in carbohydrate oxidation was associated with the observed reduction in power output (r = 0.64, 95% CI, 0.01, 0.91, *P* = 0.05) and augmented sweat rates (r = 0.85, 95% CI, 0.49, 0.96, *P* = 0.002). Plasma HSP70 and adrenaline concentrations were not increased with exercise in either environment.

**Conclusion:**

These data contribute to our understanding of how moderate environmental heat stress is likely to influence substrate oxidation and plasma HSP70 expression in an ecologically-valid model of endurance exercise.

## Introduction

During endurance exercise, carbohydrates and fatty acids are the primary substrates oxidised to support the adenosine triphosphate (ATP) turnover required for repeated skeletal muscle contraction (Hawley and Leckey 2015; O’Brien et al. 1993; Watt et al. 2002). The absolute and relative contributions made by these substrates to total energy expenditure is largely determined by exercise intensity and duration (Jeukendrup et al. 1998; Romijn et al. 1993; Watt et al. 2002). Additionally, environmental heat stress increases carbohydrate and reduces fat oxidation during exercise at given work rates (Febbraio et al. 1994a, b; Jentjens et al. 2002; Hargreaves et al. 1996a). This shift in substrate utilisation may be explained by heat-stress-induced increases in core and muscle temperatures (Febbraio et al. 1994a, b; Fernández-Elías et al. 2015), dehydration (Wilson et al. 1977; Febbraio et al. 1996, 1998), and increases in circulating adrenaline concentrations (González-Alonso et al. 1999; Hargreaves et al. 1996b; Powers et al. 1985). The metabolic response to exercise has implications for fatigue (Bergström and Hultman 1967), the adaptive response to training (Philp et al. 2012), and refuelling requirements (Impey et al. 2018).

Exposure to environmental heat stress during endurance exercise also increases intracellular and extracellular heat shock protein 72 (HSP72) concentrations (Gibson et al. 2014; Magalhães et al. 2010; Marshall et al. 2006; Morton et al. 2006; Périard et al. 2012; Whitham et al. 2007). Intracellular HSP72 acts as a molecular chaperone that accompanies misfolded and denatured proteins to maintain cellular homeostasis (Whitham and Fortes 2008), and subsequently contributes to the development of thermotolerance (Li et al. 1995; Liu et al. 1992). In skeletal muscle, this chaperoning function of HSP72 may contribute to mitochondrial adaptations to endurance exercise training (Henstridge et al. 2014; Skidmore et al. 1995; Young et al. 2003). Mechanistically, HSP72 is released into the circulation through the α_1_-adrenoceptor pathway, and is therefore stimulated by circulating catecholamines (Johnson et al. 2005; Whitham et al. 2006), which are increased by exercise (Febbraio et al. 2002a; Walsh et al. 2001). During stress, the elevation of extracellular HSP72 expression may act a ‘danger signal’ to prime or enhance immunologic responses (Fleshner and Johnson 2005; Fleshner et al. 2003). It is possible that HSP72 responses are influenced by exercise in a duration-and-intensity dependent manner (Fehrenbach et al. 2005; Liu et al. 1999).

Previous research has studied these stimulatory effects of environmental heat stress on substrate metabolism (Maunder et al. 2020; Febbraio et al. 1994a; Jentjens et al. 2002; Young et al. 1985; Marino et al. 2001) and HSP72 expression (Gibson et al. 2014; Yamada et al. 2007; Walsh et al. 2001; Whitham et al. 2006) during endurance exercise at matched external work rates between-environments. In the real-world, however, endurance athletes are likely to experience a reduction in absolute work rates when training in hot vs. temperate conditions (Boynton et al. 2019; Lorenzo et al. 2010; Maunder et al. 2021a, b). Given that research supports the importance of exercise intensity in both substrate utilisation (Lorenzo et al. 2010; Boynton et al. 2019; Maunder et al. 2021a, b) and HSP72 expression (Gibson et al. 2014; Morton et al. 2009), it is possible that these acute effects of environmental heat stress may be at least partially blunted by the likely reduction in absolute work rates. Therefore, a relevant comparison for practitioners considering the likely metabolic effects of performing a training session under environmental heat stress may be using matched relative physiological stress, or heart rates.

Accordingly, the primary aim of the present investigation was to assess the effects of moderate environmental heat stress on substrate oxidation rates during heart rate-matched moderate-intensity cycling. Additionally, we investigated the response of plasma HSP70 to the exercise protocols. We hypothesised that lower rates of whole-body energy expenditure and carbohydrate oxidation would be observed under moderate environmental heat stress secondary to lower achieved power outputs, but that plasma HSP70 expression would still be elevated due to greater increases in core temperature and plasma adrenaline concentrations.

## Methods

### Ethical approval

This study was performed in accordance with the standards of the Declaration of Helsinki, 2013. The Auckland University of Technology Ethics Committee approved all procedures (21/121), and all participants provided written informed consent prior to participation. This study was not registered in a database. Data associated with this study are available from the corresponding author upon reasonable request.

### Participants

Ten endurance-trained male cyclists and triathletes participated in this study (age, 31 ± 8 years; height, 181 ± 3 cm; body mass, 75.0 ± 5.7 kg; peak oxygen uptake [${\dot{\text{V}}\text{O}}_{{{\text{2peak}}}}$], 58.1 ± 6.8 mL·kg^−1^·min^−1^; first ventilatory threshold [VT_1_], 204 ± 46 W; overall training volume, 9 ± 3 h·wk^−1^; cycling volume, 6 ± 2 h·wk^−1^). Participants were free from viral infection (> 1 month), lower-limb injury (> 3 months), and had not suffered with any cardiovascular disease, or previously experienced exertional heat stress illness. Participants were unacclimated to exercise-heat stress, which was defined as not recently having undertaken specific heat acclimatisation training (≥ 6 months). Using 50% of the between-group effect size for post-exercise extracellular HSP72 concentration (Whitham et al. 2007), it was a priori calculated that a total sample size of 10 participants would be required to observe a between-group difference (*P* < 0.05) in post-exercise extracellular HSP72 concentration with 80% statistical power. This smaller effect size was utilised as smaller between-environment differences in post-exercise thermoregulatory variables were expected in this study (~ 1 °C difference in T_re_ based on Maunder et al. (2020), compared to the ~ 2 °C difference in Whitham et al. (2007)).

### Study design

**Fig. 1:**
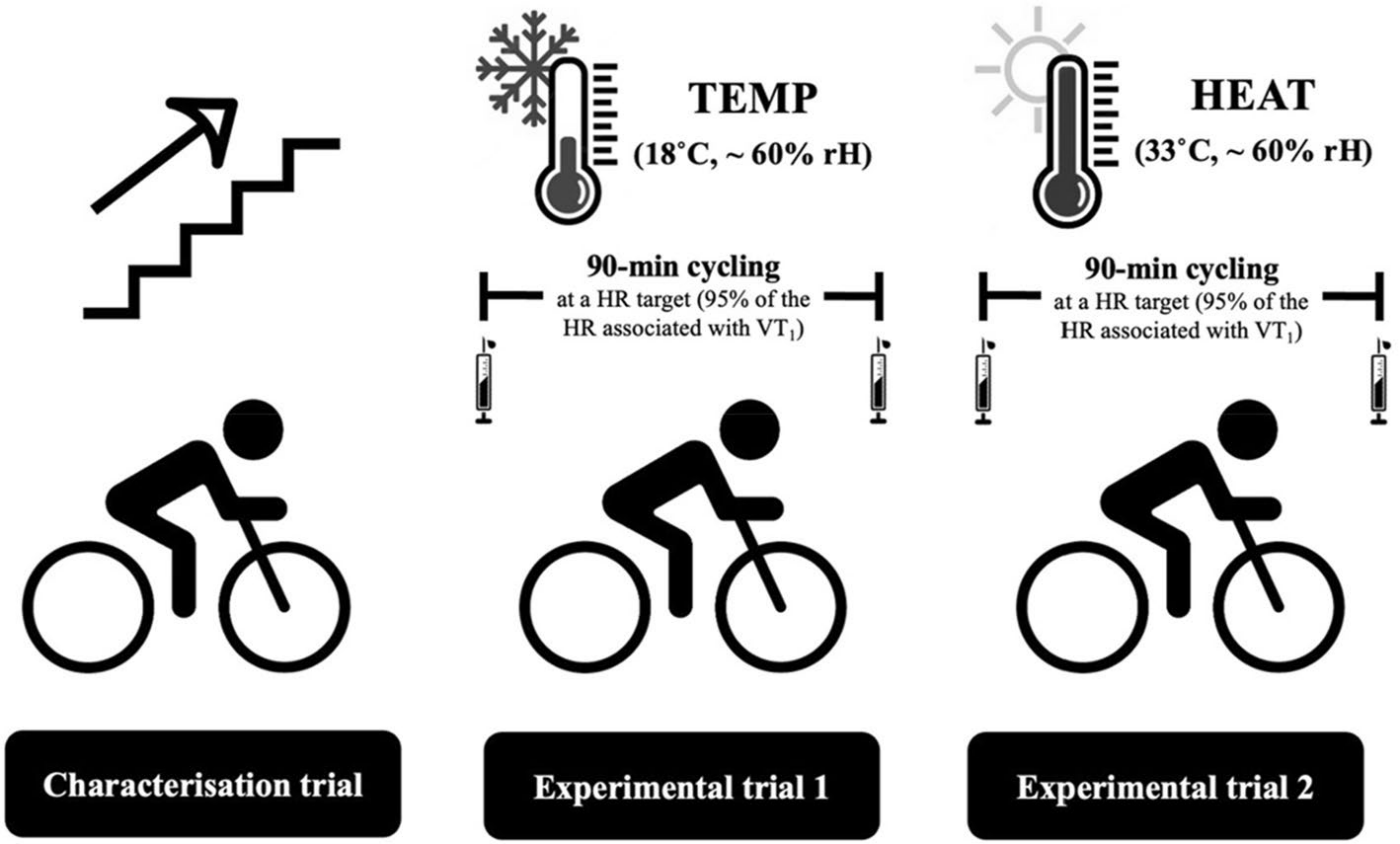
Experimental overview

An acute, randomised, counterbalanced cross-over design was used in the present investigation. Participants visited the laboratory on three occasions, ~ 7 d apart, for: (i) a characterisation trial involving an incremental cycling test, and (ii) two experimental trials, which each involved a 90-min bout of cycling exercise at 95% of the HR associated with VT_1_ in either 18 (TEMP) or 33 °C (HEAT), with ~ 60% relative humidity (rH) (Fig. 1). The order in which participants completed the two experimental trials was randomised with a counterbalanced design. All laboratory trials were intended to be completed during the non-summer months in Auckland, New Zealand. However, data collection was interrupted by a nationwide lockdown for COVID-19. Consequently, four participants completed their trials during the winter months, and six of the ten participants were tested during the warmer months. Therefore, subgroup analyses were performed to assess seasonal effects on the primary outcome measures of this study.

### Characterisation trial

Participants arrived the laboratory at ~ 7:00 am for the characterisation trial following an overnight fast, having refrained from caffeine, alcohol, and intense exercise for 24 h. On arrival, participants provided written informed consent and completed a health screening questionnaire. Height and body mass were first measured. Participants then mounted a laboratory ergometer (Excalibur Sport, Lode, Groningen, NET), and an incremental cycling test commenced at 95 W in a laboratory environment (18 °C , 60–80% rH). The power output was increased by 35 W every 3 min until the respiratory exchange ratio reached 1.0, after this point the duration of each stage was shortened to 1 min until task failure. Expired gases were collected throughout using a metabolic cart (TrueOne2400, ParvoMedics, Sandy, UT, US). ${\dot{\text{V}}\text{O}}_{{{\text{2peak}}}}$ was determined to ensure eligibility for study participation of ${\dot{\text{V}}\text{O}}_{{{\text{2peak}}}}$  ≥ 50 mL·kg^−1^·min^−1^, and VT_1_ was also determined to individualise the exercise intensity in the experimental trials. HR was also measured continuously using a chest-strap HR monitor (TICKR, Wahoo, Taiwan). The ${\dot{\text{V}}\text{O}}_{{{\text{2peak}}}}$ was identified as the highest 30-s average value for ${\dot{\text{V}}\text{O}}_{{2}}$ during the incremental cycling test. The VT_1_ was identified as the first increase in the ventilatory equivalent for oxygen (${\dot{\text{V}}\text{E}}\cdot{\text{VO}}_{{2}}^{{ - {1}}}$) without changes in ventilatory equivalent for carbon dioxide (${\dot{\text{V}}\text{E}}\cdot{\text{VCO}}_{{2}}^{{ - {1}}}$) (Lucía et al. 2000), and expressed as both a power output and HR value. Convective air flow was provided using a pedestal fan (GCPF340, Goldair, China).

### Experimental trials

Participants arrived at the laboratory ~ 7 d following the characterisation trial at ~ 7:00 am, having adhered to the same pre-trial instructions described above, and made written records of their diet for 48 h and training for 7 d, such that these could be repeated in advance of the second experimental trial. On arrival, a 6-mL pre-exercise blood sample was drawn from an antecubital vein using the venepuncture technique. Pre-exercise body mass was measured in cycling clothes and then participants self-inserted a rectal thermometer ∼10 cm beyond the anal sphincter in privacy for determination of rectal temperature (T_re_). A skin temperature thermistor was subsequently taped over the mid-belly of the vastus lateralis ∼15 cm above the patella, and covered with two pieces of 2-mm neoprene in order to insulate the skin underneath for continuous observation of insulated skin temperature (T_ins_), which was used for estimation of muscle temperature (T_mus_) in line with recent work (Flouris et al. 2015). Participants then sat comfortably for 5 min for measurement of resting estimation T_mus_ and T_re_ in the laboratory environment before entering the environmental chamber, which was set at either 18 or 33 °C , with 60% rH. Participants then mounted their own road bicycle, which was connected to a calibrated, direct-drive indoor trainer (Kickr, Wahoo Fitness, Atlanta, USA), and were fitted with a chest-strap HR monitor (TICKR, Wahoo, Taiwan) for continuous observation of HR during exercise.

A 90-min bout of cycling then commenced. During the first 5-min of cycling, participants were asked to progressively increase their HR to a specified target equivalent to 95% (± 2 b·min^−1^) of the VT_1_ HR determined in the incremental cycling test, and then maintain their target HR until exercise cessation. Participants were reminded of this if their HR drifted outside the target range. Participants had ad libitum access to plain water throughout the cycling trial. Convective air flow (~ 3.2 m·s^−1^) was provided by an industrial fan (FS-75, FWL, Auckland, NZ). Expired gases were obtained for 4 min every 15 min using a metabolic cart (TrueOne2400, ParvoMedics, Sandy, UT, US). Rating of perceived exertion (RPE) on a scale of 6 to 20 (Borg 1982) was recorded every 15 min. Perceived thermal comfort on a 1-to-10 scale, and thermal sensation on a 1-to-14 scale, which were adapted from an previous work (Gagge et al. 1967), were also assessed every 15 min. The T_re_ was monitored throughout the cycling trial to ensure that it did not exceed 39.5 °C (Silva et al. 2019), and no participant reached this temperature.

Within 3-min after exercise cessation, a 6-mL post-exercise antecubital venous blood sample was obtained using venepuncture technique, and total water consumption was recorded through weighing of drink bottles. Participants subsequently removed the HR monitor, skin temperature thermistor, and rectal thermometer in privacy. Participants dried their skin using a towel, and post-exercise body mass was measured in cycling clothes for calculation of percentage dehydration (Eq. 1), and estimation of the magnitude of sweat loss during the trial, accounting for fluid consumption. Participants returned to the laboratory ~ 7 d later to perform the remaining experimental trial, having adhered to the same pre-trial instructions described above, and repeated their 48-h diet and 7-day training records in advance.

1 $${\text{Percentage dehydration }}\left( \% \right) \, = \, \left[ {\left( {{\text{Body mass}}_{{{\text{pre}} - {\text{exercise}}}} {-}{\text{ Body mass}}_{{{\text{post}} - {\text{exercise}}}} } \right) \div {\text{Body mass}}_{{{\text{pre}} - {\text{exercise}}}} } \right] \, \times { 1}00$$

Equation 1 Calculation of percentage dehydration (%).

### Expired gas analyses

In the incremental cycling test, V̇O_2_ and V̇CO_2_ from the last 1 min of each stage were used to estimate substrate oxidation rates using standard stoichiometric equations (Eq. 2) (Jeukendrup and Wallis 2005). Peak fat oxidation rate (g·min^−1^) was then identified as the highest fat oxidation rate during the test. In the experimental trials, expired gases from the final three minutes of each 4-min sampling time point were averaged and used for calculation of whole-body rates of energy expenditure, carbohydrate oxidation, and fat oxidation using standard equations (Eq. 2). The first minute of each sampling timepoint was discarded to minimise any potential hyperventilatory effect associated with reorganising the headgear, and to ensure that participants had resettled into their comfortable cycling position. The rate of metabolic heat production (H_prod_) was calculated by subtracting external mechanical power output (W) from metabolic energy expenditure (W) (Ravanelli et al. 2020). The H_prod_ is expressed relative to body surface area according to the Dubois and Dubois formula (Du Bois and Du Bois 1916).

2a $${\text{Energy expenditure }}\left( {{\text{EE}}} \right)\left( {{\text{kcal}}\cdot{\text{min}}^{{ - {1}}} } \right) \, = \, \left( {0.{55}0 \, \times {\dot{\text{V}}\text{CO}}_{{2}} } \right) \, + \, \left( {{4}.{471 } \times {\dot{\text{V}}\text{O}}_{{2}} } \right)$$

2b $${\text{Carbohydrate oxidation }}\left( {{\text{CHO}}} \right)\left( {{\text{g}}\cdot{\text{min}}^{{ - {1}}} } \right) \, = \, \left( {{4}.{21}0 \, \times {\dot{\text{V}}\text{CO}}_{{2}} } \right) \, {-} \, \left( {{2}.{962 } \times {\dot{\text{V}}\text{O}}_{{2}} } \right)$$

2c $${\text{Fat oxidation }}({\text{g}}\cdot{\text{min}}^{{ - {1}}} ) \, = \, \left( {{1}.{695 } \times {\dot{\text{V}}\text{O}}_{{2}} } \right) \, {-} \, \left( {{1}.{7}0{1 } \times {\dot{\text{V}}\text{CO}}_{{2}} } \right)$$

Equation 2 Estimates of whole-body rates of energy expenditure, carbohydrate oxidation, and fat oxidation, where both carbon dioxide production (${\dot{\text{V}}\text{CO}}_{{2}}$), and oxygen uptake (${\dot{\text{V}}\text{O}}_{{2}}$) are in L·min^−1^.

### Thermoregulatory analyses

Mean values obtained from the rectal thermometer and skin temperature thermistor during the 30-s prior to each measurement timepoint were defined as T_re_ and T_ins_. The T_ins_ was used for estimation of the *vastus lateralis* temperature (T_mus_) in line with previous work (Eq. 3) (Flouris et al. 2015).

3a $$\mathrm{Estimated }\,\,{\mathrm{T}}_{\mathrm{mus}}\mathrm{ at rest }\left(\mathrm{^\circ{\rm C} }\right) = \left(0.597\times {\mathrm{T}}_{\mathrm{ins}}\right) - \left(0.439\times {\mathrm{T}}_{\mathrm{ins}}{\mathrm{Lag}}_{2}\right) + \left(0.554\times {\mathrm{T}}_{\mathrm{ins}}{\mathrm{Lag}}_{3}\right) - \left(0.709\times {\mathrm{T}}_{\mathrm{ins}}{\mathrm{Lag}}_{4}\right)+ 14.767$$

3b $$\mathrm{Estimated }\,\,{\mathrm{T}}_{\mathrm{mus}}\mathrm{ during\,\, exercise }\,\, \left(\mathrm{^\circ{\rm C} }\right) = \left(0.599\times {\mathrm{T}}_{\mathrm{ins}}\right) - \left[\left(0.311\times {\mathrm{T}}_{\mathrm{ins}}{\mathrm{Lag}}_{4}\right)\right] + 15.63$$

Equation 3 Estimates of insulated skin temperature over the vastus lateralis (T_ins_) at rest and during exercise, where T_ins_Lag_2_ = T_ins_ – T_ins_ two min beforehand, etc.

### Plasma analyses

Venous blood samples were stored on ice in pre-chilled ethylenediaminetetraacetic acid tubes until trial completion. In order to allow the correction of plasma concentrations for changes in plasma volume across the trial (van Beaumont et al. 1972), a small sample of whole blood was pipetted into duplicate capillary tubes before spinning for 3-min using a micro-haematocrit centrifuge (Haematospin 1400, Hawksley& Sons, Ltd., England), and then the proportion of red blood cells was manually measured (the coefficient of variation [CV], 1.55 ± 1.85%). Plasma was then extracted from the remaining whole blood by centrifugation in 4 °C for 10 min using a *Heraeus Megafuge 16* Centrifuge (D-37520 Osterode, Thermo Fisher Scientific, Inc., Germany), and stored at − 80 °C for further analyses. Plasma HSP70 concentration was determined via an enzyme-linked immunosorbent assay (ELISA) (BMS2087, Thermo Fisher Scientific, Inc., US). Plasma adrenaline and noradrenaline concentrations were determined via ELISA kits (ab287788 and ab287789, Abcam^®^, UK). These assays were performed using commercially available kits in duplicate according to the manufacturer’s instructions. Achieved intra-assay CVs were 13.0% for plasma HSP70 concentration, 4.3% for plasma adrenaline concentration, and 16.6% for plasma noradrenaline concentration.

### Statistical analyses

Statistical analysis was performed with GraphPad Prism Version 9.3.1 (GraphPad Software, San Diego, CA, USA). Data are presented as mean ± standard deviation (SD) unless otherwise stated. The normality of data distributions were assessed using the Shapiro–Wilk test, which is considered as an appropriate test for small sample sizes (N < 50) (Rahman and Govindarajulu 1997). Within- and between-trial differences in physiological variables were assessed using two-way, repeated measures analyses of variance, with temperature and time as factors. Simple comparisons were made using paired t-tests (or the Wilcoxon signed-rank test for non-normal distributions). Where main effects of time were observed, exercise-induced changes (15 vs. 90 min) in variables were compared. Where main effects of environmental temperature were observed, end-exercise values were compared. Where interactions between trial and time were observed, the magnitude of exercise-induced changes (15 vs. 90 min) in variables were compared. Bivariate linear correlations (Pearson’s product-moment correlation coefficients or Spearman’s rank-order correlation coefficients, depending on the distribution of the data) were used to assess relationships between primary outcome measures (heat stress-induced changes in mean carbohydrate oxidation rate and post-exercise plasma HSP70 concentrations) and heat stress-induced changes in dehydration percentage, sweat rate, mean power output, mean T_re_, and mean estimated T_mus_. The level of statistical significance was set at *P* ≤ 0.05.

## Results

**Fig. 2:**
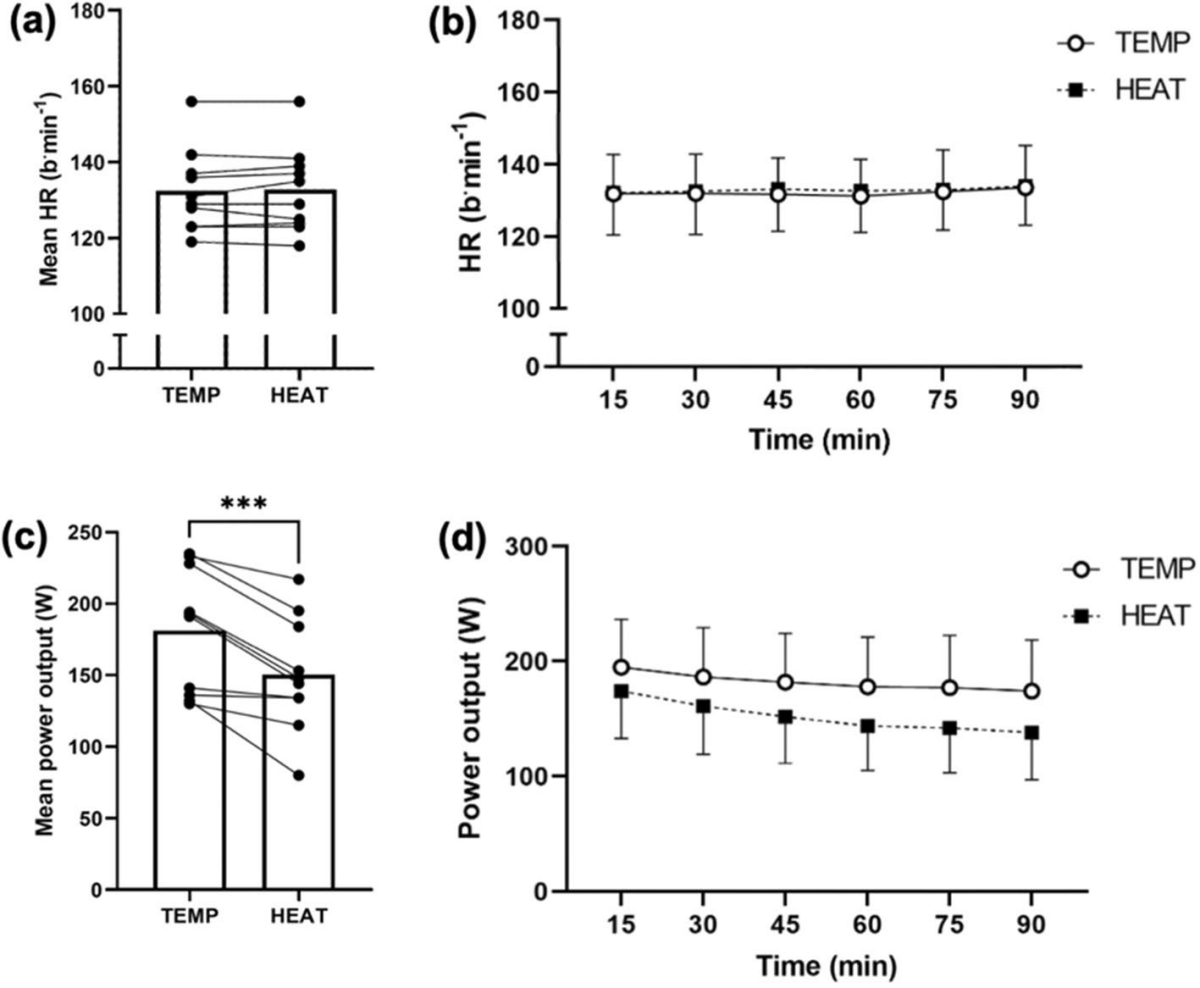
Heart rate (HR, beats·min^−1^) and power output (W) during 90 min of moderate-intensity exercise at a target HR of 95% of the first ventilatory threshold in 18 °C (TEMP) and 33 °C (HEAT). **a** Mean HR during the 90-min exercise trials (beats·min^−1^), **b** HR at each timepoint throughout the 90-min exercise trials (beats·min.^−1^), **c** mean power output (W) during the 90-min exercise trials, and **d** power output (W) at each timepoint throughout the 90-min exercise trials. Bars indicate group mean values, and dots identify individual mean values. ***Indicates *P* ≤ 0.001

Mean HR was not significantly different between TEMP and HEAT (*P* = 0.63, Fig. 2a); in all cases, the individual difference in mean HR between-trials was ≤ 4 b·min^−1^. Although a main effect of time for HR was observed (*P* = 0.01), there was no main effect of environmental temperature (*P* = 0.23), or interaction between time and environmental temperature (*P* = 0.53, Fig. 2b). Subgroup analyses indicated no significant effect of season for mean whole-body EE (*P* = 0.54), CHO oxidation rate (*P* = 0.58), fat oxidation rate (*P* = 0.78), or exercise-induced change in post-exercise plasma HSP70 concentration (*P* = 0.26).

Mean power output was significantly lower in HEAT (17 ± 11%, *P* < 0.001, Fig. 2c); in all participants, mean power output was numerically lower in HEAT (5–64 W). A main effect of time was observed for power output, whereby power output significantly decreased as exercise progressed (*P* < 0.001, Fig. 2d). A significant interaction between time and environmental temperature was observed (*P* = 0.01, Fig. 2d). The reduction in power output over time (15 vs. 90 min) was greater in HEAT (22 ± 11 vs. 11 ± 7%, *P* = 0.005). Accordingly, the rate of metabolic heat production was lower in HEAT vs. TEMP (308 ± 77 vs. 354 ± 84 W·m^−2^, *P* < 0.001).

**Fig. 3:**
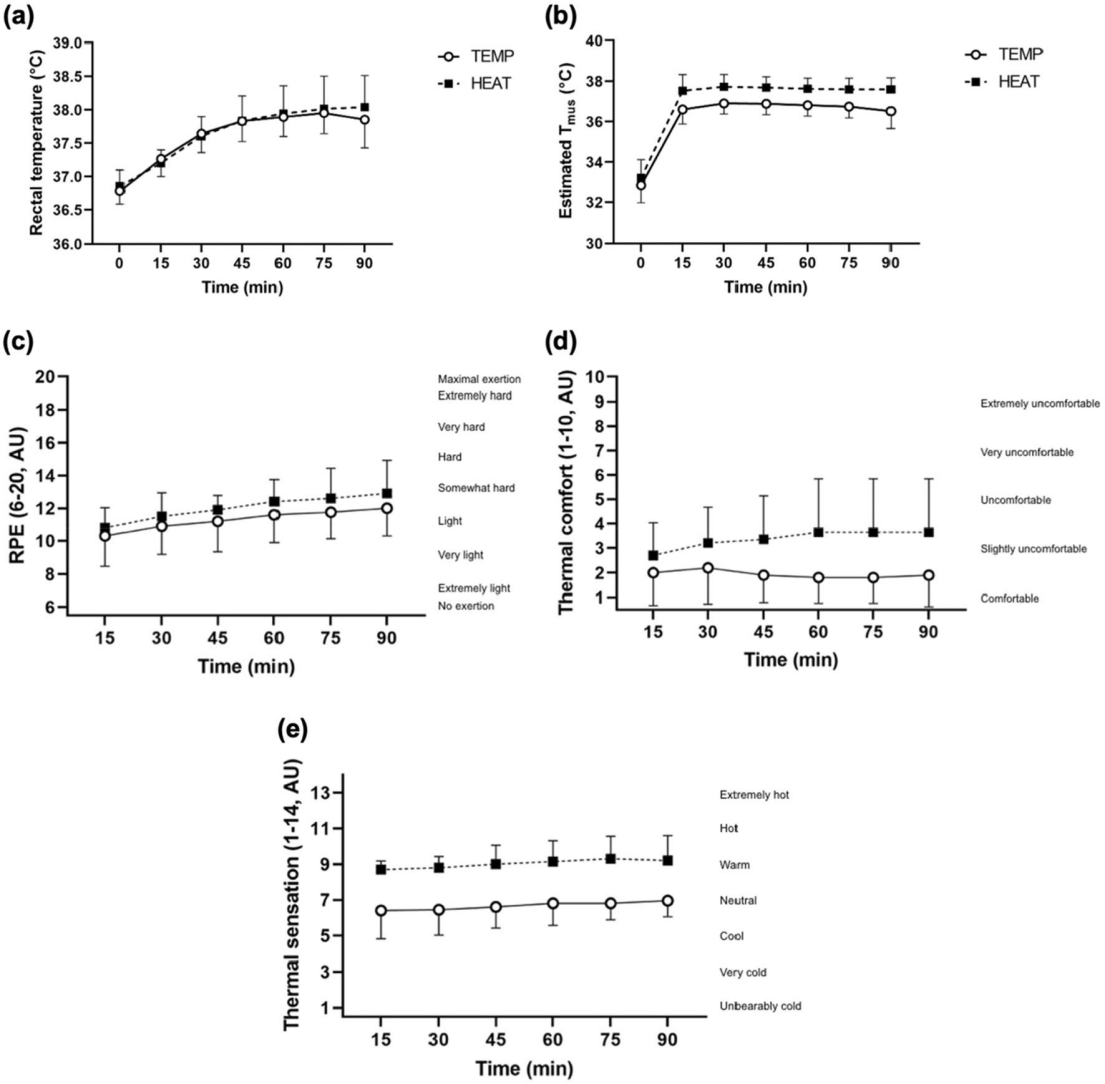
Thermoregulatory and perceptual variables. **a** Rectal temperature (°C ), **b** estimated muscle temperature (°C ), **c** rating of perceived exertion (6–20, AU), **d** thermal comfort (1–10, AU), and **e** thermal sensation (1–14, AU) at each timepoint during 90 min of moderate-intensity exercise with a target HR of 95% of the first ventilatory threshold in 18 °C (TEMP) and 33 °C (HEAT). A solid line with clear markers indicates data obtained from TEMP, and a dashed line with solid markers indicate data obtained from HEAT

Both T_re_ and estimated T_mus_ significantly increased as exercise progressed (T_re_, 1.1 ± 0.4 and 1.2 ± 0.6 °C ; estimated T_mus_, 3.8 ± 0.7 and 4.5 ± 0.9, in TEMP and HEAT, respectively, from rest to 90 min, both *P* < 0.001, Fig. 3a, b). No main effect of environmental temperature (*P* = 0.49), or interaction between time and environmental temperature was observed for T_re_ (*P* = 0.58, Fig. 3a). A main effect of environmental temperature was observed for estimated T_mus_, whereby the end-exercise estimated T_mus_ was greater in HEAT (1.0 ± 0.4 °C , *P* < 0.001, Fig. 3b). A significant interaction between time and environmental temperature was observed (*P* = 0.017). The increase in estimated T_mus_ from rest to 90 min was greater in HEAT (4.5 ± 0.9 vs. 3.8 ± 0.7 °C , *P* = 0.003). Sweat rate (1.1 ± 0.4 vs. 0.6 ± 0.3 L·h^−1^, *P* < 0.001) and dehydration (1.4 ± 0.6 vs. 0.9 ± 0.7% of pre-exercise body mass, *P* = 0.003) were significantly greater in HEAT.

A main effect of time for RPE was observed, whereby it significantly increased as exercise progressed (*P* < 0.001, Fig. 3c). There was no significant main effect of environmental temperature (*P* = 0.09), or interaction between time and environmental temperature (*P* = 0.76) for RPE. No main effect of time was observed for thermal comfort (*P* = 0.12), but a significant effect of environmental temperature was observed, whereby thermal comfort was significantly higher in HEAT (*P* = 0.001, Fig. 3d). A significant interaction between time and environmental temperature was observed (*P* = 0.03), whereby participants felt less comfortable as exercise duration progressed in HEAT. No main effect of time was observed for thermal comfort (*P* = 0.10), but a significant effect of environmental temperature was observed, whereby thermal sensation was significantly higher in HEAT (*P* < 0.001, Fig. 3e). No interaction between time and environmental temperature interaction was observed for thermal sensation (*P* = 0.89).

**Fig. 4:**
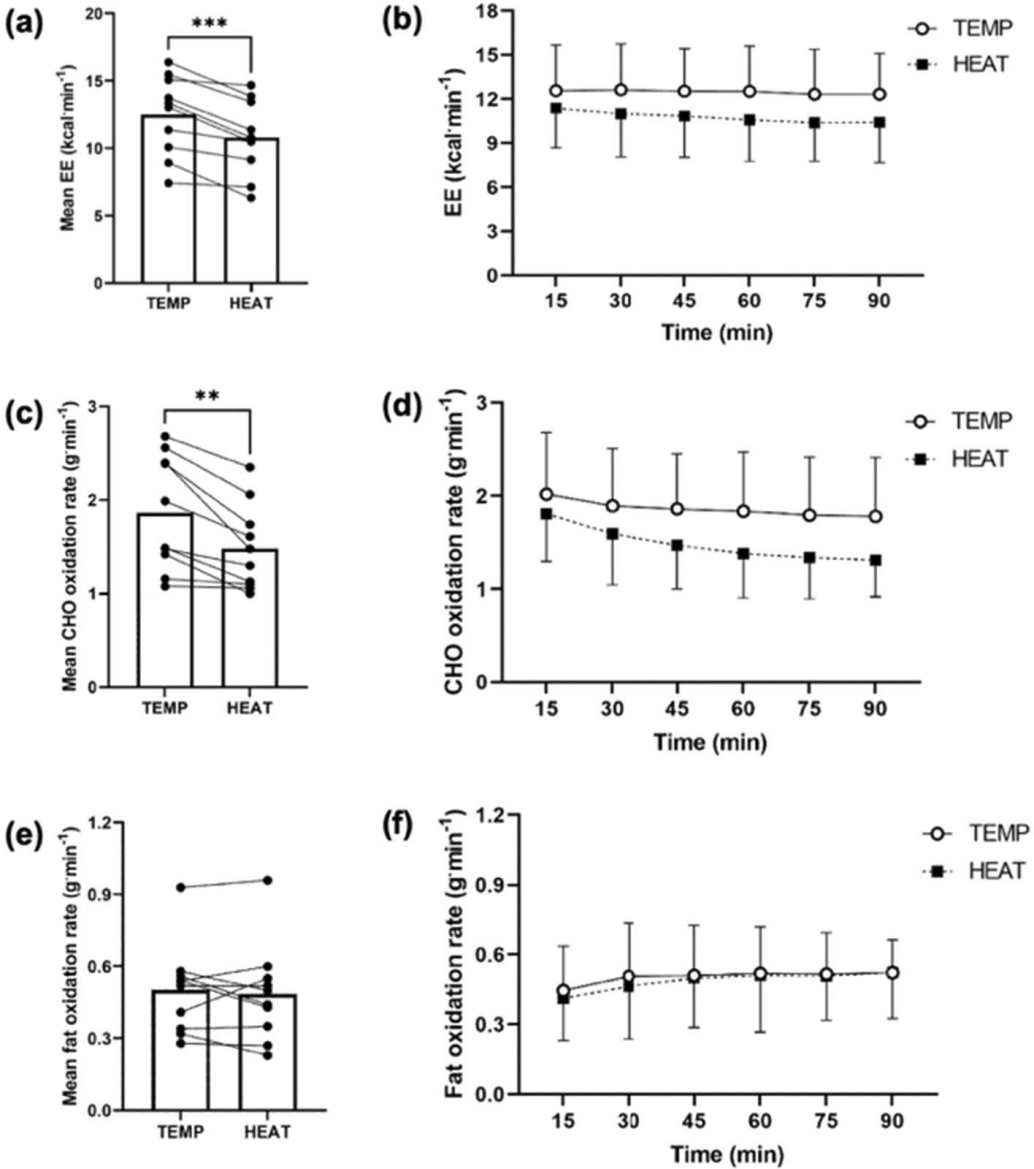
Whole-body substrate oxidation rates during 90 min of moderate-intensity exercise with a target HR of 95% of the first ventilatory threshold in 18 °C (TEMP) and 33 °C (HEAT). **a** Mean energy expenditure (kcal·min^−1^), **b** energy expenditure (kcal·min^−1^) at each 15-min timepoint, **c** mean carbohydrate oxidation rate (g·min^−1^), **d** carbohydrate oxidation rate (g·min^−1^) at each 15-min timepoint, **e** mean fat oxidation rate (g·min^−1^), **f** fat oxidation rate (g·min.^−1^) at each 15-min timepoint. Bars indicate group mean values, and dots identify individual mean values. ***Indicates *P* ≤ 0.001. **Indicates *P* ≤ 0.01

Mean whole-body EE was significantly lower in HEAT (14 ± 8%, *P* < 0.001, Fig. 4a). A main effect of time was observed for whole-body EE (*P* = 0.002), whereby it significantly decreased as exercise progressed (12.0 ± 2.9 vs. 11.4 ± 2.9 kcal^.^min^−1^, at 15 vs. 90 min, respectively, *P* = 0.03), but there was no interaction between time and environmental temperature (*P* = 0.13, Fig. 4b). Mean CHO oxidation rate was significantly lower in HEAT (19 ± 11%, *P* = 0.002, Fig. 4c). A main effect of time was observed for CHO oxidation rate, whereby it significantly decreased as exercise progressed (*P* = 0.003, Fig. 4d). A significant interaction between time and environmental temperature was observed (*P* = 0.02). The exercise-induced reduction in CHO oxidation rate over time (15 vs. 90 min) was significantly greater in HEAT (27.1 ± 10.1 v. 9.6 ± 21.3%, *P* = 0.003, Fig. 4d). Mean fat oxidation rate was not significantly different between-trials (*P* = 0.54, Fig. 4e). A main effect of time was observed for fat oxidation (*P* < 0.001), whereby it significantly increased as exercise progressed (0.43 ± 0.18 and 0.52 ± 0.17 g·min^−1^, at 15 and 90 min, *P* = 0.001, Fig. 4f). No main effect of temperature (*P* = 0.54), or interaction between time and environmental temperature (*P* = 0.73), was observed for fat oxidation rate.

**Fig. 5:**
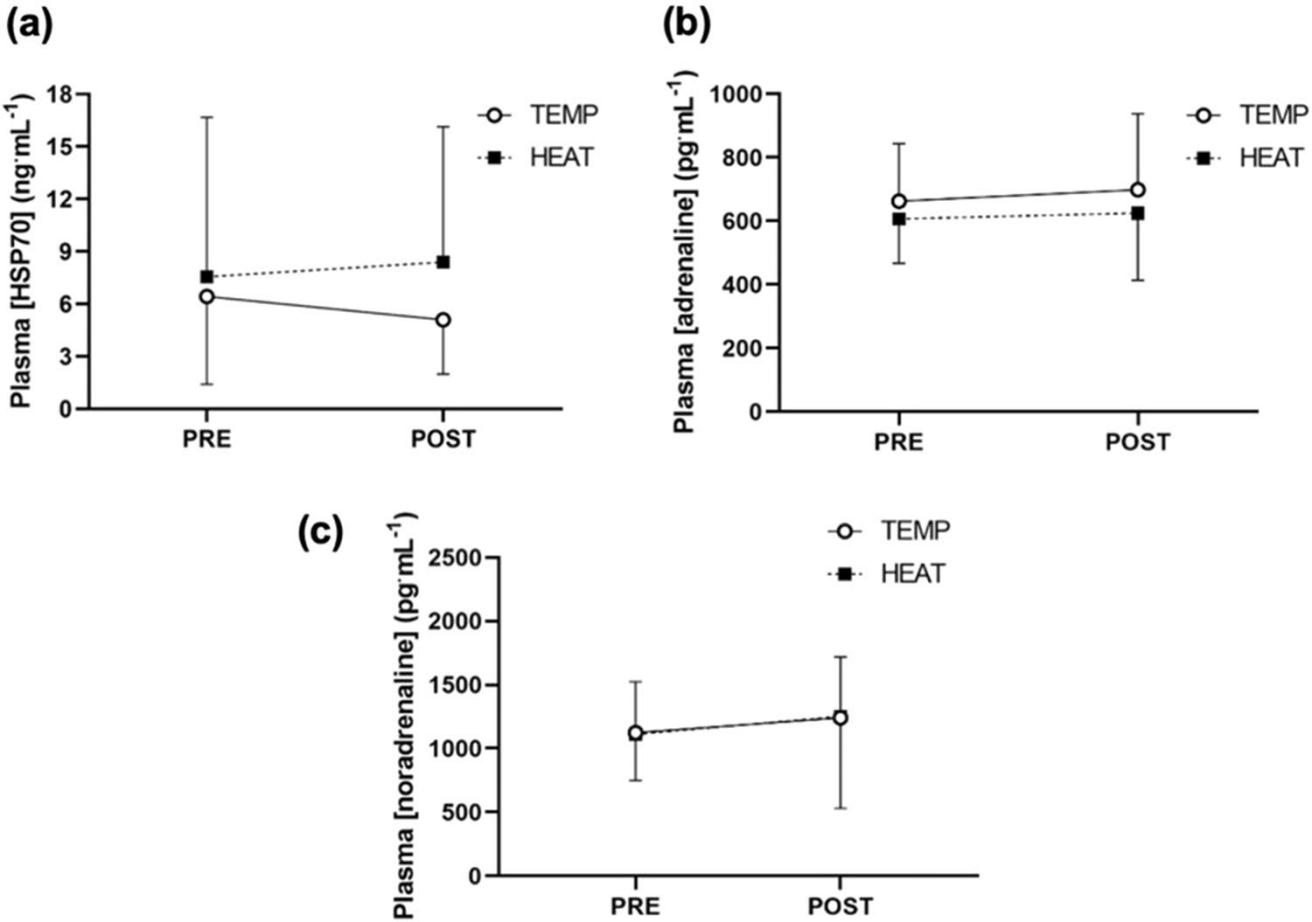
Mean pre- and post-exercise plasma. **a** Heat shock protein 70 (ng·mL^−1^) (TEMP, N = 8; HEAT, N = 9), **b** adrenaline (pg·mL^−1^) (N = 9), and **c** noradrenaline (pg·mL^−1^) (N = 10) concentrations in 18 °C (TEMP) and 33 °C (HEAT). A solid line with clear markers indicates data obtained from TEMP, and a dashed line with solid markers indicate data obtained from HEAT

No main effect of time (*P* = 0.80), environmental temperature (*P* = 0.51), or interaction between time and environmental temperature (*P* = 0.30), was observed for plasma HSP70 concentration (Fig. 5a). No main effect of time (*P* = 0.65), environmental temperature (*P* = 0.31), or interaction between time and environmental temperature (*P* = 0.86), was observed for plasma adrenaline concentration (Fig. 5b). No main effect of time (*P* = 0.23), environmental temperature (*P* = 0.98), or interaction between time and environmental temperature (*P* = 0.91), was observed for plasma noradrenaline concentration (Fig. 5c).

Heat stress-induced in changes in mean CHO oxidation rate were significantly correlated with heat stress-induced changes in power output (r = 0.64, *P* = 0.05), and sweat rate (r = 0.85, *P* = 0.002). No significant correlations were observed between heat stress-induced changes in post-exercise plasma HSP70 concentration and heat stress-induced changes in mean power output, dehydration, sweat rate, mean T_re_, or mean estimated T_mus_ (Table 1).

**Table 1:** Bivariate linear correlations between primary outcome measures (heat stress-induced changes in mean carbohydrate oxidation rate and post-exercise plasma HSP70 concentration) and input variables (heat stress-induced changes in mean power output, dehydration, sweat rate, mean rectal temperature, and mean estimated muscle temperature)

	Δ Mean CHO oxidation rate (g·min^−1^)	Δ Post-exercise plasma HSP70 concentration (ng·mL^−1^)
	(N = 10)	(N = 8)
Δ Mean power	**r = 0.64**	r_s_ = − 0.45
(W)	**(0.01, 0.91)**	(− 0.88, 0.37)
	***P* = 0.05**	*P* = 0.27
Δ Dehydration	r = 0.42	r_s_ = − 0.19
(% of body mass)	(− 0.29, 0.83)	(− 0.79, 0.59)
	*P* = 0.23	*P* = 0.66
Δ Sweat rate	**r = 0.85**	r_s_ = − 0.40
(L·h^−1^)	**(0.49, 0.96)**	(− 0.86, 0.42)
	***P* = 0.002**	*P* = 0.33
Δ Mean T_re_	r = − 0.37	r_s_ = 0.17
(°C )	(− 0.83, 0.39)	(− 0.61, 0.78)
	*P* = 0.33*	*P* = 0.69
Δ Mean estimated T_mus_	r = 0.08	r_s_ = 0.60
(°C )	(− 0.58, 0.68)	(− 0.19, 0.92)
	*P* = 0.83	*P* = 0.13

Data are expressed with 95% confidence intervals

Δ represents the difference between the two experimental trials (HEAT – TEMP)

*CHO oxidation* Carbohydrate oxidation, *HSP70* Heat shock protein 70, *T*_*re*_ Rectal temperature, *estimated T*_*mus*_ Estimated muscle temperature, *r* Pearson’s product-moment correlation coefficients, *r*_*s*_ Spearman’s rank-order correlation coefficients

*Missing data due to technical difficulty (N = 9)

Significant correlations are in bold

## Discussion

The purpose of the present investigation was to assess the effect of moderate environmental heat stress on substrate oxidation rates and plasma HSP70 expression in response to heart rate-matched prolonged moderate-intensity cycling. The primary findings were that (i) whole-body CHO oxidation rates were lower, but whole-body fat oxidation rates were similar, during moderate-intensity cycling in HEAT vs. TEMP, with these effects likely due to the reduction in power output and associated decrease in EE in HEAT, and (ii) moderate-intensity cycling did not increase plasma HSP70 expression in either HEAT or TEMP.

In this present study, exercise intensity was regulated in relation to HR thresholds (95% of the HR associated with VT_1_), and therefore, the reduced power output in HEAT vs. TEMP was not unexpected. Indeed, a decrease in mean power output was observed in all participants in HEAT (− 17 ± 11% from mean power output in TEMP, *P* = 0.001, Fig. 2c). Lower power output achieved during endurance exercise—at a given HR – in HEAT may be explained by increased peripheral blood flow to regulate temperature via sweating (Febbraio 2001). Greater peripheral blood flow in HEAT is supported by the observed elevated sweat rate in HEAT (1.1 ± 0.4 vs. 0.6 ± 0.3 L·h^−1^, *P* < 0.001). Greater demand for peripheral blood flow necessitates greater cardiac output and reduced stroke volume via the Frank-Starling mechanism, and therefore results in an increase in HR during exercise at given absolute work rates (Trinity et al. 2010). Thus, participants had to reduce power output to maintain the HR target in HEAT.

In alignment with our hypothesis, whole-body CHO oxidation rates were lower in HEAT vs. TEMP (1.48 ± 0.46 vs. 1.86 ± 0.61 g·min^−1^, *P* = 0.002, Fig. 4c). These findings contrast previous research demonstrating increased carbohydrate metabolism during endurance exercise performed under environmental heat stress (≥ 30 °C ) compared to equivalent exercise in temperate conditions at matched external work rates, where core temperatures were greater in the environmental heat stress trials and higher than in the present study (Febbraio et al. 1994a, b; Jentjens et al. 2002; Hargreaves et al. 1996a; Maunder et al. 2020). We took this approach in order to describe physiological responses to an ecologically valid scenario. The lack of difference in core temperature can be explained by the lower H_prod_ in HEAT vs. TEMP (308 ± 77 vs. 354 ± 84 W·m^−2^, *P* < 0.001), secondary to the lower EE and power output.

The bivariate correlational analyses indicate the observed heat stress-induced decrease in whole-body CHO oxidation rates may be explained by the associated reduction in power output in HEAT (Table 1). The lower absolute power output in HEAT reduced whole-body EE (− 14 ± 8% from mean EE in TEMP, *P* < 0.001, Fig. 4a), and therefore the demand for ATP production via carbohydrate metabolism. Interestingly, whole-body fat oxidation rates were unaffected by environmental temperature (*P* = 0.54, Fig. 4e). Therefore, the observed heat stress-induced reduction in whole-body EE associated with the lower power output during moderate-intensity cycling performed in HEAT is explained by reduced CHO oxidation and maintained fat oxidation rates. However, the addition of an experimental trial in HEAT with the exercise intensity at absolute power output matched to TEMP would be necessary to confirm this.

Furthermore, the observed heat stress-induced increase in sweat rate was related to the heat stress-induced decrease in whole-body CHO oxidation rate (Table 1). Plausibly, participants who had a larger increase in sweat rate in HEAT could dissipate heat more efficiently in HEAT, and therefore produce more metabolic heat for the same T_re_ response, resulting in a more subtle reduction in power output in HEAT vs. TEMP. Therefore, athletes with greater between-environment difference in sweat rate had smaller reductions in power output, and therefore smaller reductions in CHO oxidation rates.

However, this heat stress-induced decrease in whole-body CHO oxidation rate when exercising at matched HR might not be observed in endurance athletes acclimated to hot environments. Following acclimation, athletes produce more power at a given HR under heat stress (Yamada et al. 2007; Corbett et al. 2022), likely necessitating a greater demand for ATP synthesis, and therefore CHO oxidation, than when unacclimated. Accordingly, the between-trial differences we observed here might be reduced following a period of heat acclimation training.

Secondly, moderate-intensity endurance exercise did not increase plasma HSP70 expression in either TEMP or HEAT (Fig. 5a). These findings contrast previous studies reporting a significant increase in extracellular *HSP72* concentration in unacclimated individuals following endurance exercise performed in temperate (18–24 °C ) (Whitham et al. 2007; Fehrenbach et al. 2005) and hot environments (35–40 °C ) (Magalhães et al. 2010; Marshall et al. 2006; Gibson et al. 2014; Périard et al. 2012; Whitham et al. 2007). The ELISA kit used in the present investigation quantified the concentration of the whole HSP70 family (including HSP72 and HSP73 isoforms). This could explain the greater plasma HSP70 concentrations (~ 6–9 ng·mL^−1^) observed in this present study, compared with the other studies measuring extracellular HSP72 (~ 1–6 ng·mL^−1^) (Magalhães et al. 2010; Marshall et al. 2006; Whitham et al. 2007; Périard et al. 2012; Fehrenbach et al. 2005). As HSP73 is unlikely to be stimulated by environmental heat stress (Kregel 2002; Locke 1997; Welch and Suhan 1986), it is plausible that the capture of the whole HSP70 may have obscured an effect of endurance exercise on plasma HSP72 expression. However, the lack of observed plasma HSP70 accumulation might also be explained by the absence of exercise-induced increase in plasma adrenaline concentration (Fig. 5b), given catecholamines stimulate the release of HSP72 into the circulation (Chin et al. 1996; Heneka et al. 2003).

The extracellular HSP72 response to endurance exercise is related to exercise intensity and duration (Fehrenbach et al. 2005), with the addition of internal thermal stress promoting further increases extracellular HSP72 responses (Marshall et al. 2006). In this present investigation, expired gases collected during TEMP indicated the exercise intensity corresponded to 60.7 ± 5.5%${\dot{\text{V}}\text{O}}_{{{\text{2peak}}}}$. This is relatively similar to studies reporting increased concentrations of plasma HSP72 after endurance exercise intensity prescribed at ~ 60% ${\dot{\text{V}}\text{O}}_{{{\text{2peak}}}}$ (Whitham et al. 2007; Fehrenbach et al. 2005), but may have been insufficient in our cohort to induce increased extracellular HSP70 concentration. Similarly, insufficient exercise duration may explain the lack of stimulation of extracellular HSP70, as the aforementioned studies included longer exercise protocols (90 vs. 120 min) (Whitham et al. 2007; Fehrenbach et al. 2005). A longer exercise duration may further increase heat stress-induced physiological strain relevant to HSP72 accumulation, which may include elevated core and/or local temperatures (Morton et al. 2006; Cuthbert et al. 2019), or decreased carbohydrate availability (Dalgaard et al. 2022; Febbraio and Koukoulas 2000; Febbraio et al. 2002b). Plausibly, an exercise-induced increase in plasma HSP70 expression may have been observed in this present study if exercise duration was ≥ 120 min. Given HEAT did not increase T_re_ or plasma adrenaline concentrations vs. TEMP, it is unsurprising that a stimulatory effect of heat stress on plasma HSP70 expression was not observed.

From a practical standpoint, our descriptive data might be used to help inform nutrition practices in endurance training. Our data suggests that when endurance athletes are exposed to a hot environment during training, either incidentally (e.g. during summer months) or deliberately (e.g. heat acclimatisation training), the carbohydrate and overall energy cost of exercise might be reduced compared to training performed at a similar HR in temperate conditions.

In conclusion, heart rate-matched moderate-intensity cycling in a hot environment (33 °C ) reduced whole-body CHO oxidation rates, but did not alter fat oxidation rates, compared to the equivalent exercise performed in a temperate environment (18 °C ). This metabolic change was associated with the lower external power output in HEAT. Exercise-induced increases in plasma HSP70 expression did not occur in either condition. These data should be considered by practitioners working with endurance athletes who are exposed to moderate environmental heat stress, either incidentally or purposefully, during training and/or competition.

## Data Availability

Data is available from the corresponding author upon reasonable request.

## Code availability

Code is available upon reasonable request.
